# Low uptake of Aboriginal interpreters in healthcare: exploration of current use in Australia’s Northern Territory

**DOI:** 10.1186/s12913-017-2689-y

**Published:** 2017-11-15

**Authors:** Anna P. Ralph, Anne Lowell, Jean Murphy, Tara Dias, Deborah Butler, Brian Spain, Jaquelyne T. Hughes, Lauren Campbell, Barbara Bauert, Claire Salter, Kylie Tune, Alan Cass

**Affiliations:** 1 Menzies School of Health Research, Charles Darwin University, Darwin, NT Australia; 2 Royal Darwin Hospital, Darwin, NT Australia; 3 Research Centre for Health and Wellbeing, Charles Darwin University, Darwin, NT Australia; 4 Aboriginal Interpreter Service, Darwin, NT Australia; 5 Office of Aboriginal Health Policy & Engagement, Department of Health, Northern Territory Government, Darwin, NT Australia

## Abstract

**Background:**

In Australia’s Northern Territory, most Aboriginal people primarily speak an Aboriginal language. Poor communication between healthcare providers and Aboriginal people results in adverse outcomes including death. This study aimed to identify remediable barriers to utilisation of Aboriginal Interpreter services at the Northern Territory’s tertiary hospital, which currently manages over 25,000 Aboriginal inpatients annually.

**Methods:**

This is a multi-method study using key stakeholder discussions, medical file audit, bookings data from the Aboriginal Interpreter Service 2000–2015 and an online cross-sectional staff survey. The Donabedian framework was used to categorise findings into structure, process and outcome.

**Results:**

Six key stakeholder meetings each with approximately 15 participants were conducted. A key structural barrier identified was lack of onsite interpreters. Interpreter bookings data revealed that only 7603 requests were made during the 15-year period, with completion of requests decreasing from 337/362 (93.1%) in 2003–4 to 649/831 (78.1%) in 2014–15 (*p* < 0.001). Non-completion was more common for minority languages (p < 0.001). Medical files of 103 Aboriginal inpatients were audited. Language was documented for 13/103 (12.6%). Up to 60/103 (58.3%) spoke an Aboriginal language primarily. Of 422 staff who participated in the survey, 18.0% had not received ‘cultural competency’ training; of those who did, 58/222 (26.2%) indicated it was insufficient. The Aboriginal Interpreter Service effectiveness was reported to be good by 209/368 (56.8%), but only 101/367 (27.5%) found it timely. Key process barriers identified by staff included booking complexities, time constraints, inadequate delivery of tools and training, and greater convenience of unofficial interpreters.

**Conclusion:**

We identified multiple structural and process barriers resulting in the outcomes of poor language documentation and low rates of interpreter bookings. Findings are now informing interventions to improve communication.

**Electronic supplementary material:**

The online version of this article (10.1186/s12913-017-2689-y) contains supplementary material, which is available to authorized users.

## Background

When cultural difference or language discordance is present between healthcare providers and clients, poor communication can occur, with major adverse consequences [1, 2]. High-quality intercultural communication is a core component of ‘cultural security’ or ‘cultural competence’, defined as ‘awareness of the cultural factors that influence another’s views and attitudes, and an assimilation of that awareness into professional practice’ [3]. Better intercultural communication can be achieved through the use of professional interpreters, who can have beneficial impacts on comprehension, uptake of health care, clinical outcomes and satisfaction with care [1, 4, 5]. Effective training in cultural competence can also have benefits for improved patient care [6, 7]. Good communication is integral to the provision of safe, high-quality care, and facilitates shared decision-making which in turn promotes better health outcomes [8].

In Australia, Indigenous peoples (Aboriginals and Torres Straight Islanders) are 2.3 times as likely to die early or live with poor health as non-Indigenous Australians [9], and a 10-year mortality gap persists between Indigenous and non-Indigenous Australians [10]. In Australia’s Northern Territory (NT), Indigenous people comprise 30% of the population, and an estimated 60% speak an Indigenous language at home [11]. Royal Darwin Hospital is the main tertiary referral hospital in the NT. The number of Aboriginal dialects actively spoken in the hospital’s catchment and referral area is around 50, with mutual comprehension between some. Ineffective communication between healthcare providers and Indigenous clients is highly prevalent [12–17]. Documented adverse outcomes include: refusing treatment due to misunderstanding, resulting in death; [2] consenting to surgery without knowing what the operation entails; [18] fundamental misunderstanding, confusion and frustration; [12, 19] unnecessarily prolonged admission; [18] taking leave from hospital against medical advice [20, 21] and distrust of healthcare providers with the belief that information is being deliberately withheld [14, 16].

While many people whose first language is an Aboriginal language have conversational English skills, an interpreter is still highly valuable for complex medical communication, decision-making and for helping to mitigate alienating medical environments [12, 22]. When conversational English skills are present, patients and healthcare providers can both underestimate the extent of miscommunication [12]. A study at Royal Darwin Hospital in 2004 documented that despite an Aboriginal Interpreter Service being established in the year 2000, utilisation remained low [15].

Language discordance can be compounded by discordance in health beliefs and world view more broadly such that it may take hours or days of discussion with the patient and an interpreter before a shared understanding is reached [18]. Furthermore, medical history-taking, entailing detailed and repeated questioning, can be confronting, insulting and baffling for traditional Aboriginal people [18].

In order to inform the design of an intervention to improve communication, we sought to identify current practices in inter-cultural communication between healthcare providers and Aboriginal patients at Royal Darwin Hospital. Our aims were to identify remediable barriers to interpreter use, by ascertaining: [1] trends in Aboriginal Interpreter Service bookings data, and [2] healthcare provider knowledge, attitudes and practices regarding cultural competency training, use of interpreters and documentation of language. In parallel with this, an in-depth qualitative arm of the study is underway comprising interviews with key informants about inter-cultural communication [23]. Although findings from the NT may be unique to this setting, general principles of quality and safety are broadly applicable to other healthcare settings caring for Indigenous people, or indeed any non-dominant language groups.

## Methods

This is a multi-method study utilising information derived from discussions with key stakeholders, data audited from medical files, retrospective data on Aboriginal Interpreter Service bookings, and an online cross-sectional staff survey. The Donabedian evaluation model [24] which describes factors impacting on quality of healthcare in terms of structure, process and outcome, was used as the framework to categorise findings. In this study, ‘outcome’ relates to interim outcomes relevant to interpreter access, not patient health outcomes. The study setting was Royal Darwin Hospital (Top End Health Services), the Northern Territory’s tertiary referral hospital where Aboriginal patients comprise approximately 54% of the patient population [25]. Royal Darwin Hospital utilises the NT Aboriginal Interpreter Service, established in 2000 to service territory-wide health, legal and government agencies.

### Key stakeholder discussions

Regular investigator meetings comprising a broad group of relevant stakeholders including the listed authors (healthcare providers, Aboriginal Interpreter Service employees, the hospital nurse consultant for Aboriginal consumer engagement, academics and policy makers) were held throughout the project to ensure a broad understanding of issues relating to intercultural communication and interpreter use. Meeting minutes provided data on structure and process factors relevant to the evaluation.

### Aboriginal interpreter service hospital bookings

Top End Health Services bookings data for each financial year from 2000 to 2015 were provided by the Aboriginal Interpreter Service. This database includes information on all referrals for an interpreter for a Top End Health Services client including Royal Darwin Hospital inpatients, outpatients and boarders, as well as requests from outside Royal Darwin Hospital (clinics, other Northern Territory hospitals). Relevant variables included date of request, job status (Completed or Cancelled [‘Interpreter did not show’ or ‘No Interpreter available’]); an identifier for the person requiring the interpreting service; whether the job was a rostered job or not; location of job request and language required. A minority language was defined for the purposes of this analysis as one for which <15 requests were made during the 15-year period.

### Documentation of language

Preferred language (language spoken at home) is documented for adult inpatients at Royal Darwin Hospital by nursing staff on a paper form (Multi-disciplinary Admission / Discharge Tool). The form is a comprehensive medical admission document for each patient that includes fields to be completed on language spoken at home, requirement for an interpreter and whether an interpreter booking has been made. Documentation was ascertained in a point-prevalence survey by identifying all available medical files of adult Aboriginal inpatients on a single day (22 December 2015). Inclusion criteria were: adult patients identified in clerking software to be Aboriginal, admitted to general or specialist medical or surgical wards including the rapid assessment planning unit, Coronary Care, Geriatrics and Renal wards. Patients in the Intensive Care, Psychiatric or Maternity wards were excluded for logistic reasons. Information pertaining to language and interpreter requirement was recorded in an excel database.

### Staff survey

Staff at Royal Darwin Hospital were invited via email to participate in an online survey (Additional file 1). The email was addressed to clinical staff, but non-clinical staff were included since many (e.g. ward clerks) have face-to-face interactions with Aboriginal patients, and others (e.g. nursing administration positions) have past relevant clinical experience. For data analyses, healthcare providers were defined as nurses, doctors and allied health staff (physiotherapist, speech pathologist, occupational therapist, social worker, dietician, hearing screener, orthoptist and ‘allied health not otherwise specified’). The survey asked about cultural competency training, experiences communicating with Aboriginal people and experiences of using the Aboriginal Interpreter Service. Suggestions for improvement were sought. The survey was open for 9 weeks from July–September 2016; an initial invitation and two reminders were sent. An incentive (a chance to win movie tickets) was provided and posters displayed prominently around the hospital provided information about the survey.

### Statistical analyses

Analyses were undertaken in Stata 14.1 (College Station, Texas 77,845 USA). Figures were created in GraphPad Prism 5.0 (GraphPad Software Inc., La Jolla, California). Descriptive statistics were used, chi-squared test for comparison of independent proportions, and chi-squared test for trend for proportions over 15 years. Sample size calculations were not undertaken for this observational study. For consistency, percentages are provided to 1 decimal place throughout despite varied sample sizes. Qualitative data (free text comments from the staff survey) were subjected to thematic analysis using inductive coding.

## Results

Findings of this multi-method study are summarised according to the Structure, Process, Outcome framework in Table 1 [24], and presented below according to the data collection method employed.

**Table 1:** Evaluation of the use of Aboriginal Interpreters according to the Structure, Process, Outcome model

FACTOR IMPACTING ON INTERPRETER UPTAKE	STRUCTURE		PROCESS		OUTCOME	
	Description	Data source	Description	Data source	Description	Data source
Service set-up	-Hospital and interpreter services are in separate locations and have different governance	-Key stakeholder discussions	-Interpreter booking procedure can be complex -Coordination of health provider activities around interpreter availability is difficult	-Staff survey -Staff survey	-Few interpreter bookings were made -21.2% of bookings were not fulfilled.	-AIS database -AIS database
Tools and training	-Cultural competency training is mandatory	-Key stakeholder discussions	-Training provided during orientation is brief. Not all staff receive the training	-Staff survey	-29.7% of staff were not satisfied with the cultural competence training received	-Staff survey
	-A tool to determine who needs an Aboriginal interpreter exists	-Key stakeholder discussions	-Awareness of the tool among healthcare providers is very low	-Staff survey	-31.6% of staff lacked confidence in determining who requires an interpreter	-Staff survey
Documentation	-Language is meant to be documented in a nursing admission form	-Key stakeholder discussions	-There is poor documentation and a lack of familiarity among healthcare providers with Aboriginal language names	-Medical file audit	-Aboriginal language was documented for only 12.6% of patients audited	-Medical file audit
Use of unofficial interpreters	-Hospital policy discourages the use of unofficial interpreters (e.g. ‘escorts’ or family members)	-Key stakeholder discussions	-Ease of access to unofficial compared with trained interpreters means unofficial interpreters are commonly used	-Staff survey	-44.3% of staff reported that they often use an unofficial interpreter	-Staff survey

### Key stakeholder discussions

Up to 15 individuals, mostly represented in the author list, participated in six investigator meetings between June 2015 and October 2016. Key findings obtained via these key stakeholder discussions, relevant to the ‘structure’ category in the evaluation model, comprised knowledge about existing policies, tools and training available for healthcare providers, and mechanisms for accessing interpreters. Specifically, we ascertained that Royal Darwin Hospital does not employ on-site interpreters; the Aboriginal Interpreter Service is located a 20-min drive away and provides an on-demand service via a bookings system plus a rostered service whereby one language interpreter is seconded to the hospital four hours per weekday. Interpreter bookings are chiefly made by a third party (hospital-based Aboriginal Liaison Officers) rather than the healthcare provider seeking an interpreter. Training in Aboriginal cultural competency, including a focus on intercultural communication, is mandatory for hospital-based healthcare providers. A tool to help healthcare providers decide if their client requires an interpreter, developed by the Aboriginal Interpreter Service, is circulated during staff orientation in a session presented by the Aboriginal Interpreter Service. Hospital policy states that untrained interpreters such as family members of patient ‘escorts’ should not be used to provide medical interpretation.

### Aboriginal interpreter service hospital bookings

**Fig. 1:**
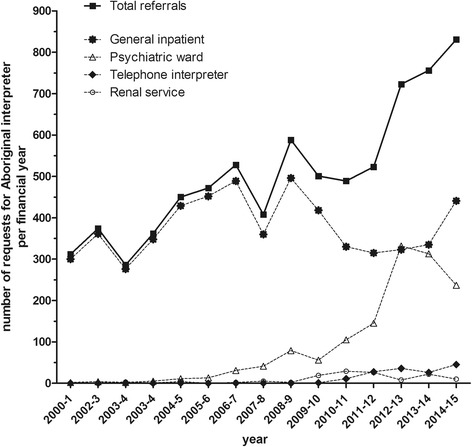
Number of Aboriginal Interpreter Service bookings made by Top End Health Services, June 2000–June 2015, showing breakdown by main service areas

Investigation of the interpreter bookings database provided results relevant to the ‘outcome’ category of the evaluation model (Table 1). There were 7603 requests for an Aboriginal interpreter for Top End Health Services clients June 2000–June 2015. Annual documented referrals increased from 312 in 2000–1 to 831 in 2014–5 (Fig. 1), during which time a similar percentage increase in inpatient numbers occurred (from approximately 10,000 to 22,000 non-haemodialysis Indigenous patient admissions annually (Jean Murphy, personal communication and health service annual report [25]). The upward trend in booking requests was largely accounted for by increases attributed to the psychiatric ward and to the cancer care centre (established 2010), rehabilitation and renal services, and use of telephone interpreters (Fig. 1).

**Fig. 2:**
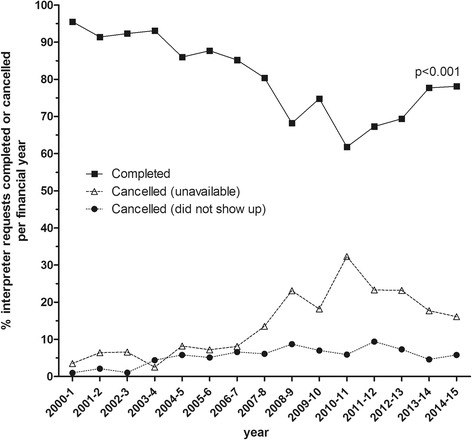
Outcome of requests for an Aboriginal interpreter

**Fig. 3:**
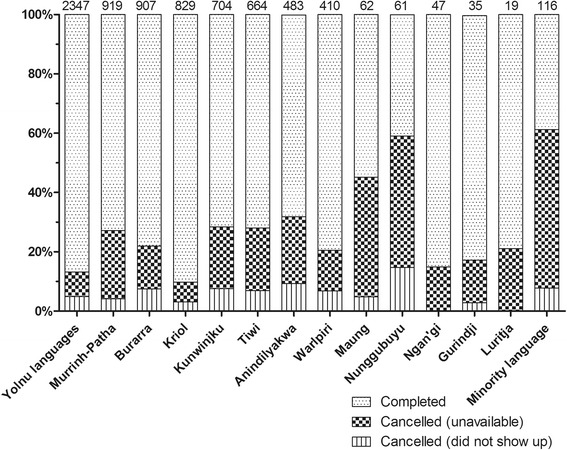
Outcome of interpreter booking according to language requested *Numbers at tops of columns indicate total number of requests for that language*

Interpreter requests were made for 46 Aboriginal languages or dialects during the 15-year period. Overall, 5988/7603 (78.8%) of requests were completed, with a downward trend over time shown in Fig. 2, from 337/362 (93.1%) in 2003–4 to 649/831 (78.1%) in 2014–15 (χ^2^ test for trend: *p* < 0.001). Reasons provided in the Aboriginal Interpreter Service bookings database for non-completion were ‘no interpreter available’ or ‘interpreter did not show’ (Fig. 2). Bookings for minority languages were significantly less likely to be completed (45/116, 38.8%) compared with more commonly-spoken Aboriginal languages (e.g. Yolŋu languages: 2038/2347, 86.8%; p < 0.001; Kriol 748/829, 90.2%; p < 0.001), although higher completion rates were achieved for some less commonly-requested languages (e.g. Gurindji) (Fig. 3).

**Fig. 4:**
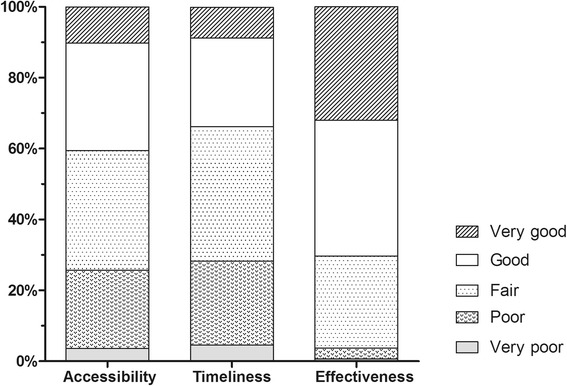
Staff perceptions of Aboriginal Interpreter Service accessibility, timeliness and effectiveness

Investigation of individual interpreter bookings for the 2014–5 year showed that of 831 bookings, 699 were for 378 patients (245 single interpreting sessions, 133 repeat bookings), and the remaining 321 bookings were documented as being for ‘various community members’, the Royal Darwin Hospital general roster or for making a health educational video.

### Audit of language documentation

One hundred and three Aboriginal inpatients were identified as eligible for inclusion in the Multi-disciplinary Admission / Discharge Tool audit (Tables 1 and 2). Main language spoken at home was blank in 34 (33.0%) of ‘completed’ forms. 26 (25.2%) patients were documented to speak an Aboriginal language; however, the name of an Aboriginal language was accurately provided - permitting spelling mistakes - in only 13 (12.6%) instances. Two patients were noted to require an interpreter; in both instances, ‘Main language spoken at home’ was blank. There was no documentation of an interpreter booking being made for any patient among the files audited. Therefore, at least 25.2% but up to 58.3% (including those left blank) of Aboriginal patients spoke an Aboriginal language at home. The 58.3% estimate is similar to that provided by the Australian Bureau of Statistics (60%) [11]. In 2014–5, based on approximate Royal Darwin Hospital inpatient numbers (22,000) [25], this would comprise around 12,800 people.

**Table 2:** Documentation of language

	Number	Percentage
Number of files audited	103	
Document available in file	103	100%
Main language spoken at home		
Blank	34	33%
English	40	39%
Aboriginal language*	26	25%
N/A or ‘unable to communicate’	3	3%
Interpreter needed		
Blank	41	40%
Yes	2	2%
No	60	58%
Interpreter booking documented		
Blank	91	88%
Yes	0	0%
No	12	12%

*Language or dialect not specified in 13 instances (given as ‘Aboriginal’); specified in 13 instances: Anindilyakwa, Djambupunyu, Duwala, Gumatj, Kriol, Tiwi, Warlpiri, Yolŋu

### Staff survey

Findings from the staff survey chiefly addressed ‘process’ and ‘outcome’ categories of the evaluation model (Table 1). Of 4067 people who received the invitation to participate in the survey, 422 responded (Tables 3 and 4). Respondents comprised 41.5% nurses, 20.9% doctors, 13.2% allied health staff, 5.0% administrative staff, 3.3% pharmacists or pharmacy assistants, 1.7% Indigenous Liaison Officers, 14.7% others e.g. chaplain, counsellor, clinical photographer, scientist/technician). Most (96.8%) nominated English as their preferred language and had trained in Australia (78.8%).

**Table 3:** Staff survey - demographic information

	Number (%)
Total number of respondents	422
Role	
Nurse	175/422 (41.5)
Doctor	88/422 (20.9)
Allied Health professional	55/422 (13.0)
Pharmacist or pharmacy assistant	14/422 (3.3)
Administration including ward clerk	21/422 (5.0)
Indigenous Liaison Officer	7/422 (1.7)
Other	62/422 (14.7)
How long have you worked at Royal Darwin Hospital?	
< 1 years	75/422 (17.8)
1–2 years	64/422 (15.2)
3–10 years	168/422 (39.8)
> 10 years	115/422 (27.3)
Please specify your preferred language	
English	367/379 (96.8)
Australian Indigenous language	1/379 (0.3)
Other (Arabic, African language, Cantonese, European language, Hindi, Malay, Punjabi, Tamil)	11/379 (2.9)
Please specify your ethnicity	
Australian non-Aboriginal / non-Torres Strait Islander	262/376 (69.3)
Australian Aboriginal or Torres Strait Islander	17/376 (4.5)
British	17/376 (4.5)
Indian	13/376 (3.4)
New Zealand European	9/376 (2.4)
Chinese	8/376 (2.1)
Other	50/376 (13.3)
Where did you undertake your primary healthcare degree?	
Australia	298/378 (78.8)
New Zealand	14/378 (3.7)
Elsewhere	66/378 (17.5)

**Table 4:** Staff survey – cultural competency and interpreter use

	Number (%)
Have you participated in ‘cultural competency’ training?	
Yes	346/422 (82.0)
No	56/422 (13.3)
Don’t know / don’t remember	20/422 (4.7)
How satisfied were you with cultural competency training?	
Very dissatisfied	4/370 (1.1)
Dissatisfied	19/370 (5.1)
Neutral	87/370 (23.5)
Satisfied	157/370 (42.4)
Very satisfied	91/370 (24.6)
Don’t remember	12/370 (3.2)
Have you undertaken any of the following?	
Participated in education on Indigenous Health during undergraduate training	140/422 (33.2)
Read books/ articles about working in an Indigenous health context	284/422 (67.3)
Undertaken a higher degree course component relevant to Indigenous health	46/422 (10.9)
Learnt an Aboriginal language	34/422 (8.1)
Participated in training to work with interpreters	98/422 (23.2)
Other activity†	110/422 (23.7)
How often do you use the interpreter service?	
Never	105/396 (26.5)
Several times yearly	112/396 (28.3)
1–3 times per month	112/396 (28.3)
> =4 times per month	25/396 (6.3)
Other‡	42/396 (10.6)
How confident do you feel deciding if your patient needs an interpreter?	
Not at all confident	2/390 (0.5)
Not confident	31/390 (7.8)
Neutral	91/390 (23.3)
Confident	200/390 (51.3)
Very confident	66/390 (16.9)
How confident do you feel working with an Aboriginal interpreter?	
Not at all confident	1/390 (0.26)
Not confident	21/390 (5.4)
Neutral	91/390 (23.3)
Confident	163/390 (41.8)
Very confident	42/390 (10.8)
Don’t remember	72/390 (18.5)
Please tick any of the following that apply	
I use the Aboriginal Interpreter Service (AIS) as often as I believe I need to	204/422 (48.3)
I believe I communicate well without an interpreter	84/422 (19.9)
I would use the AIS more often but have insufficient time	91/422 (21.6)
I would use the AIS more often but is if difficult to ascertain preferred language	62/422 (14.7)
I have tried using the Aboriginal Interpreter Service but found no available interpreter	120/422 (28.4)
I would use the AIS more often but access &/or timeliness is inadequate	186/422 (44.1)
I have tried to use the AIS but the patient declined	68/422 (16.1)
I often use an escort (family member)	187/422 (44.3)
I believe the Aboriginal interpreters are not particularly helpful	8/422 (1.9)

#### Assessing whether clients require an interpreter

Overall, 63.0% of respondents reported feeling confident or very confident about assessing a patient’s need for an interpreter (clinicians: 65.4%; non-clinicians: 59.1%; *p* > 0.05), and 58.6% were confident or very confident about working with an Aboriginal interpreter (clinicians: 61.5%; non-clinicians: 52.9%; p > 0.05). Staff indicated that they did not know of the tool to help decide on the requirement for an interpreter:

*There need to be guidelines as when to use the interpreter service. Do we use interpreters for routine doctors rounds or for nursing interventions? The only time I ever see official interpreters used on the ward is when there is to be a special, scheduled family or inter-disciplinary meeting. (Registered nurse / midwife).*

#### Use of untrained interpreters

Discussion of the appropriateness and value of using non-trained family members as interpreters included both criticism and support:

*Using family members or other non professional people to conduct “*ad hoc*” interpreting is firstly a violation of privacy and we do not know how much of the correct information is actually being told to the patient and if the information we get back is valid. (Allied Health Professional).*

*Family are available and in my world typically adequate. The cost and logistics of having interpreters for every language at every point is in my view not a reality in our resource poor setting. (Consultant/Specialist).*

#### Cultural competency training

Regarding cultural competency, 82.0% of respondents recalled receiving training (clinical staff: 82.1%; non-clinical staff: 81.8%) and 248/370 (67.0%) were satisfied or very satisfied with the training. Two hundred and twenty two (52.6%) made a comment about the cultural competency training, indicating high variability in how staff rated the quality and type of training received, and 58/222 (26.2%) indicated the training received was insufficient and more time should be allocated:

*Was only one day and did not go deep enough (Consultant/Specialist).*

*It takes more than a couple of hours of induction to begin to have a grasp on these complex cultural issues. (Consultant/Specialist).*

Some commented that cultural competency training was not readily accessible:

*We were always short staffed when orientation/Cultural competency was running I could never get study leave or I had to work double shifts to attend (Social worker).*

*The aboriginal cultural awareness study day was excellent, however I had to request to do it. I feel that the people most in need of cultural competency training may not request this. It is vitally important for safe and appropriate communication. (Registered nurse / midwife).*

Others felt that the training was ‘missing the mark’:

*Orientation [*was*] short and I learned a lot more from reading books / my own investigations! (Consultant/Specialist).*

*Difficult to understand, not used on the ward, strategies that do not work were given in the orientation, out of date. (Registered nurse / midwife).*

*the cultural competency component is missing the mark…The institutional racism that exists actually is not addressed and this in my view underpins all service provision. (Counsellor).*

Some indicated that cultural competency training needed strengthening:

*seemed a little inane and broad (Registered nurse / midwife).*

#### Recognition that communication is about more than language

Of relevance to understanding process factors which impact on the quality of communication, an important theme was the recognition that communication is about more than language, .

*I think communication is just one portion…Health care professionals being more collaborative, and allowing Aboriginal patients to make choices about their health is also something that needs to be addressed. (Registered nurse / midwife).*

*I think that there are many aspects in addition to the use of interpreters thaly, unhurried manner is part of this. (Consultant /Specialist).*

#### Criticisms and suggestions to improve communication

Although staff noted the effectiveness of the Aboriginal Interpreter Service, accessibility and timeliness were rates more poorly (Fig 4). In providing free-text comments about current process barriers, a key theme included inefficiencies around identifying a given patient as needing an interpreter and ensuring an interpreter is present when required, as illustrated in the following quotations

*Availability of interpreters is grossly inadequate. The process by which an interpreter is organised is time consuming… not available in a timely fashion. Clinical jobs are very busy… The current system greatly interrupts work flow and does not encourage use of interpreters.’ (Consultant/Specialist).*

*Interpreters are often needed at short notice in theatre. it is often not recognised until they come to theatre. Theatre interpreter would be helpful however there are many languages to cover’ (Medical Officer).*

*Patients from the wards are just “left” for their outpatient appointments…99% of the time no one has flagged that the person needs an interpreter. They arrive for a 30 min appointment and then you realise you need an interpreter but by the time you can access one the appointment is over. (Allied Health Professional).*

Suggestions for improvement, provided by 30.8% of staff, are summarised in Table 5.

**Table 5:** Recommendations provided by staff in response to the survey question: ‘Please make any comments about or suggestions to improve cross-cultural communication at Royal Darwin Hospital’

THEME	QUOTATIONS PROVIDING ILLUSTRATIVE EXAMPLES
Better communication could be facilitated through improvements within the Aboriginal Interpreter Service, the interface between this service and the hospital, or having on-site interpreters,	*-Appropriately trained, health literate interpreters are an invaluable asset, and a necessary one to achieve best outcomes for our indigenous clients. More effort needs to be made to recruit, train and retain well skilled and reliable interpreters in our health service. (Registered Nurse/Midwife)* *-Would be beneficial to have more Interpreters based in the hospital…More consistency between Interpreters (some appear very experienced, some appear to not know their role) (Occupational Therapist)* *-Overall, the quality of interpreters is good...I appreciate so much having this service available. The difficulty lies in the booking system and how difficult it is to have interpreters attend at short notice. The communication from booking staff could be greatly improved. (Occupational Therapist)* *-Sometimes there are insufficient or no interpreters...There was a time I had to wait for an hour for the interpreter to show up. Sometimes cancellation of interpreter appointment was made last minute or there was a clash of appointments or a delay in appointment because of the interpreter’s previous appointment duration was extended…Overall the Interpreter service I believe is doing their best but it comes down to manpower supply and availability. (Social worker)* *-I find the Aboriginal Interpreter service is very poor. It takes me a great deal of time to book them, and I need to give them two days notice to provide an interpreter. This has a huge impact on being able to communicate important messages to patients in a timely manner. Waiting for interpreters has an impact on my estimated date of discharge. The booking system needs to be simplified, and interpreters more available. (Registered Nurse/Midwife)*
The hospital should aspire towards best practice in communication between healthcare providers and Aboriginal people	*-Zero tolerance to culturally unsafe and poor quality care. (Registered Nurse/Midwife)* *-Royal Darwin Hospital as a Centre for Excellence in Indigenous Health Training (Consultant/Specialist)* *-This is an area that should be our core business if we are serious about improving health outcomes. It seems that this area needs to be better resourced. (Speech pathologist)*
More Aboriginal health workers should be employed by the hospital	*-More aboriginal health workers please…So much better patient engagement, feel like they actually listen to me if I have an AHW with me, and the AHW can follow up without me and feedback to me any concerns the patient didn’t want to express directly to me. (Physiotherapist)*
An interpreter coordinator and/or nurse educator would facilitate better interpreter access	*-The combination of an interpreter with an indigenous health educator was particularly effective. (now no longer available). Lack of permanent onsite coordinator has also made access much more difficult. (Consultant/Specialist)*
Using peers (‘patient preceptors’) can improve communication and could be used more widely	*-It doesn’t need to be around interpreters only. The Renal Service use patient preceptor model - which helps with care navigation and help patients understand from another patient who has been through the same process. (Registered Nurse/Midwife)*

## Discussion

In this first comprehensive study of interpreter use by healthcare providers for Aboriginal patients at the Northern Territory’s largest hospital, we identified important structural and process barriers, with the resulting adverse outcome of low interpreter uptake. We found that many healthcare providers expressed a deep commitment to wanting to provide culturally appropriate care and a desire to communicate well. However, multiple barriers to effective communication were identified. Structural barriers chiefly comprised limited interpreter accessibility due to the services not being co-located geographically, with resultant lack of visibility and timeliness. Process barriers included complexity in booking interpreters, healthcare provider time constraints, inadequate delivery of tools and training in cultural competence and working with interpreters, low knowledge and documentation of what language patients speak, and preferential use of unofficial interpreters (family members) for convenience. The chief outcome identified was that only a small proportion of patients estimated to require an interpreter received access to a trained interpreter. With the estimated annual number of Indigenous inpatient admissions in 2014–5 being approximately 22,000 (Jean Murphy, personal communication and health service annual report [25]), and assuming that between 58% (as we identified) or 60% [11] speak an Aboriginal language at home, then a crude estimate is that approximately 6.5% of Aboriginal people admitted to Royal Darwin Hospital, who do not primarily speak English, have an interpreter booking made. A smaller proportion still (approximately 5.2%) have the booking completed.

A number of survey respondents indicated deeply held feelings about the topic of Aboriginal cultural competency and communication. Important concerns expressed were that cultural competency is not well achieved, more in-depth training is required, institutional racism (unconscious bias) needs to be vigorously addressed, and those most needing training may not be accessing or valuing it. Staff provided starkly opposing views on whether cultural competency in the form they received was valuable, and 18% did not recall receiving any such training despite it being mandatory. Effective training in cultural competence can improve proximal outcomes including physician practice and patient satisfaction [6], but evidence for better health outcomes is scanty; one study showed an association with improved adherence [7] but more rigorous studies in this area are needed [6, 26]. Newly-arriving staff are often unfamiliar with the vastly different medical and cultural environment of the Northern Territory compared with southern, metropolitan parts of Australia, or international settings from where they may originate - there is diverse new knowledge for staff to absorb during their orientation. Key messages about Aboriginal culture and interpreters may be swamped by ‘information overload’ during the one-off orientation.

The reason for the downward trend in proportion of interpreter requests able to be fulfilled is unclear, but may reflect undersupply of interpreters, inefficiencies in rostering and the absence of an interpreter coordinator; a position which existed at inception of the Aboriginal Interpreter Service. The low ability to provide an interpreter for minority languages identifies a particularly marginalised group. More interpreter employment, training and retention, as well as solutions such as use of telephone or audio-visual interpreting options, are required.

The perception of the Northern Territory being a resource-poor setting, as noted by one interview respondent, is telling. In fact, Australia ranks among the top 20 wealthiest nations. The concept of ‘blue marble health’, positing that neglected diseases and poor health outcomes are increasingly embedded in pockets of social deprivation within wealthy nations [27], is highly relevant in this context, and is a reminder that it should not be beyond the resources of the nation to effectively redress such problems.

Interpreter under-utilisation is not confined to this hospital or these language groups; by contrast, inadequate uptake of interpreters is emerging as a leading knowledge-practice gap nationally [28, 29] and internationally [1, 30] While healthcare professionals may acknowledge the benefit of using accredited interpreters (although 44.3% of our survey respondents reported often using a family member instead of a trained interpreter), a failure of translation into action is widely documented. In our setting, the seriousness of medical problems and the known poor health outcomes among Aboriginal people mean that better communication needs urgent prioritisation. Existing policies on Aboriginal interpreter use and tools to help clinicians determine whether their patient requires an Aboriginal interpreter, are neither known of nor implemented. This is an important process barrier to target in implementing change.

The adverse consequences of miscommunication suggest that investments in better communication are likely to be cost-effective; research to investigate cost-effectiveness is required. Re-framing best-practice communication as a health intervention which improves outcomes would be an important step in changing clinician and management mindsets about the need to adopt this practice. An obvious priority is to assist clinicians to achieve this through restructuring of interpreter access and booking processes. A key aspirational goal would be to increase staff to patient ratios in this setting, in acknowledgement of the substantial time commitment needed for effective communication.

Previous successful models trialled at Royal Darwin Hospital included employing an interpreter coordinator and a nurse educator [18] – reinstatement of these roles was nominated in the staff survey as a strategy for improvement. Current successes include the model used to achieve high interpreter uptake in the psychiatric ward, and a renal patient preceptor model which facilitates high levels of engagement and understanding for dialysis patients. Renal Preceptors are closely aligned to bicultural experts in Aboriginal culture, renal disease and local health systems, facilitating ‘safe bridges’ between patients and health care providers [31]. Successes at interstate hospitals have included development of rules for interpreter use (e.g. when to use face-to-face versus telephone interpreters) coupled with staff education, automated booking systems and measurement of interpreter-related key performance indicators for continuous quality improvement. In our setting, use of an interpreter via telephone can allow access afterhours, as well as more promptly during standard working, since the hospital and interpreter service are located in different geographical locations. Implementation of solutions is now under discussion with the Top End Health Services hospital board, which is strongly supportive of fostering best practice in communication; this will comprise the next stage of the project.

Limitations include that the audit of language documentation did not examine other fields to ascertain whether the form was poorly completed in general, or whether only specifically with regards to language fields; also, a single snapshot on a given date was undertaken, which might not be representative. However, this does not change the finding that staff caring for Aboriginal patients generally do not appear to know what language they speak. Survey respondents self-selected to participate and were predominantly Australian-born and trained; they may not represent the staff population accurately. The Top End Health Services annual report indicates that 29.0% of staff are from non-English speaking backgrounds, compared with 3.2% in our survey [25]. Limitations in the Aboriginal Interpreter Service dataset included changes over time in how variables such as location of service were recorded; however, we focused chiefly on total Aboriginal Interpreter Service bookings and avoided statistical comparisons between locations of use.

## Conclusion

Royal Darwin Hospital has a key responsibility to ensure best practice in communication, as part of a comprehensive strategy to reduce the gap in Indigenous morbidity and mortality. In-depth qualitative data currently being collected will contextualise these findings and provide greater understanding of how effective change can be implemented. This study has important implications for clinical practice and policy, with key recommendations arising which have been shared with the hospital management board. Findings are relevant for other hospitals servicing Indigenous people, and people from linguistically diverse backgrounds. Strategies which we will now seek to incorporate into a systems-level intervention include changes at provider, patient, interpreter service and whole-system levels.

## Additional file

Additional file 1: Hospital staff survey. (PDF 2.74 mb)
