# Supplementary material for: Low uptake of Aboriginal interpreters in healthcare: exploration of current use in Australia’s Northern Territory
Source: BMC Health Serv Res. 2017 Nov 15;17:733. doi: 10.1186/s12913-017-2689-y (PMC5688693; doi:10.1186/s12913-017-2689-y)
Supplement: Additional file 1: — Hospital staff survey. (PDF 2.74 mb) [file 12913_2017_2689_MOESM1_ESM.pdf]

Welcome to this survey on communication at RDH

Thank you for participating!
This should take less than 5 minutes.
Your responses will help inform strategies for improving inter-cultural communication at Royal Darwin Hospital.

Collated survey responses will be circulated back to all staff who have received the invitation to participate.

Dr Anna Ralph
on behalf of the Communicate study investigators
Staff Specialist in Infectious Diseases and General Medicine, Royal Darwin Hospital
Senior Research Fellow, Global and Tropical Health, Menzies School of Health Research

* 1. What is your current role at Royal Darwin Hospital?

* 2. How long in total have you worked at RDH, or at other hospital(s) with a high proportion of Australian Indigenous clients?

* 3. Did you receive any formal orientation when you commenced employment at RDH? *This means time set aside from clinical duties to be inducted about hospital procedures.*

* 4. Have you participated in cultural competency training at RDH either during orientation (if you received orientation), or at any time during your employment so far?

5. If yes, how satisfied were you with the cultural competency component of your orientation?

	Very dissatisfied	Dissatisfied	Neutral	Satisfied	Very satisfied	Don't remember
Satisfaction level	○	○	○	○	○	○

Please explain the reasons for your response

* 6. Have you undertaken any of the following? Tick all that apply.

- ☐ Participated in education sessions on Australian Indigenous Health during undergraduate (healthcare) degree
- ☐ Read books or articles about working in an Australian Indigenous health context
- ☐ Undertaken a higher degree course relevant to Indigenous Health (e.g. Aboriginal health module in Master of Public Health)
- ☐ Learnt an Aboriginal language
- ☐ Participated in training to work with interpreters
- ☐ Other activities you have undertaken to assist yourself in working in an Indigenous health context (please specify)

* 7. How often do you use the Aboriginal Interpreter Service for inpatients or outpatients at RDH?

8. How confident do you feel in deciding if your patient needs an interpreter?

Very confident	Confident	Neutral	Not confident	Not at all confident
○	○	○	○	○

9. How confident do you feel when you work with an interpreter from the Aboriginal Interpreter Service? *Consider your experience(s) using an interpreter to help communicate with Aboriginal patients and their families .*

Very confident	Confident	Neutral	Not confident	Not at all confident	Don't remember *or* haven't used an Aboriginal interpreter
○	○	○	○	○	○

* 10. Please select any of the following that apply to your communications with Aboriginal patients.
Tick all that apply.

- [ ] I use the Aboriginal Interpreter Service (AIS) as often as I believe I need to.
- [ ] I believe I communicate well without an interpreter.
- [ ] I would like to use the AIS more often, but I have insufficient time.
- [ ] I would use the AIS more often, but it is difficult to ascertain what the patient's preferred language is.
- [ ] I have tried to use the AIS, but found no suitable interpreter was available for the preferred language.
- [ ] I would use the AIS more often, but access and/or timeliness is inadequate.
- [ ] I have tried to use the AIS but the patient declined use of an interpreter.
- [ ] I often use an escort (e.g. family member) when interpretation is needed.
- [ ] I believe the Aboriginal Interpreters are not particularly helpful.
- [ ] Other (please specify)

* 11. Please specify your preferred language.

* 12. Please specify your ethnicity.

* 13. Where did you undertake your primary healthcare degree (e.g. BN, MBBS)?
Please do not include higher qualifications e.g. Honours, Masters or PhD. If you do not have a degree (e.g. you are an Enrolled Nurse), please specify where you undertook your highest level of education.

14. Please describe your experience of using the Aboriginal Interpreter Service.
This is a question about availability and usefulness.

	Very poor	Poor	Fair	Good	Very good	Don't know
Accessibility	○	○	○	○	○	○
Timeliness	○	○	○	○	○	○
Effectiveness (all participants had improved understanding)	○	○	○	○	○	○

If you want to make further comments about your experience of using the Aboriginal Interpreter Service, please do so here.

15. Booking an interpreter for Aboriginal patients can be done via:

- [ ] Online AIS website
- [ ] Faxing a request form
- [ ] Emailing a request form
- [ ] All of the above
- [ ] I don't know
- [ ] Other (please specify)

16. Please feel free to make any comments about or suggestions to improve cross-cultural communication at RDH.

17. Please enter your email address if you would like to enter the draw to win a prize and to receive feedback about the study. Your email address or identity will not be linked to your responses.
Responses to all survey questions will be de-identified prior to analysis.
Thank you very much for participating.

Email Address

18. If you want to provide feedback about the format of this survey, please do so here.
